# Some Objections to the Present Theories Regarding Yellow Fever

**Published:** 1880-05-20

**Authors:** T. B. Greenley

**Affiliations:** Kentucky


### Southern Medical Record.

EDITORS:

T. S. POWELL, M.D. W. T. GOLDSMITH, M.D. R. C. WORD, M.D.

*R. C. WORD, M.D., Managing Editor.*

☞All Communications and Letters on Business connected with the RECORD must be addressed to the Managing Editor.

VOL. X. ATLANTA, GA., MAY 20, 1880. No. 5.

### ORIGINAL AND SELECTED ARTICLES.

### *SOME OBJECTIONS TO THE PRESENT THEORIES REGARDING YELLOW FEVER.*

BY T. B. GREENLEY, OF KENTUCKY.

It seems that a majority of American physicians hold that yellow fever is an exotic disease, and that it never originates in the United States, but is always imported. Dr. Vanderpoel, Health Officer of the City of New York, and who occupies an eminent standing in the profession, says that Havana is the great yellow fever-breeding depot, or hot-bed from whence we derive the disease. That notwithstanding Vera Cruz and Rio Janeiro suffer from the plague, yet, as far as New York is concerned, there is little danger, from the fact that but little intercourse exists between that city and Vera Cruz; and that as Rio's summer is our winter, there is no danger from intercommunication between the two places, the disease prevailing only in hot weather. He says that Havana is probably one of the most filthy cities in the world, and on that account he would recommend the appointment of a commission by our government to act in concert with one to be appointed by the Spanish government, in order to devise such measures of a sanitary character as will not only render that city healthy, but entirely stamp out yellow fever.

The inference to be drawn from these observations is, of course, that we then would be exempt from all danger of the disease in the

United States. If this theory is true, it would be a very wise precaution, on the part of the government of Spain and our own, to appoint a commission to investigate the sanitary condition of Havana, and institute such measures as would insure improvement in its hygienic condition, without regard to pecuniary expenditure.

But the question might be asked, is this view of the origin of yellow fever correct?

As far as it applies to Havana, and being the generative product of the filthy condition of that city, I believe it is true. But I cannot see why it might not be produced in any locality under the same local sanitary and atmospheric conditions. If filth in the city of Havana can act as a cause to develop yellow fever, why should not filth in New Orleans be susceptible of producing the same result under the same atmospheric influences? It seems to me this question must be answered only in one way, and that is in the affirmative. If like causes produce like results, that is the only view the common sense mind can take of it. Until our people are convinced of the fact that filthy streets and other places in their immediate vicinity may be the means of generating the plague of yellow fever, and not rely on the preventive effects of quarantine, etc., we shall continue at intervals to be troubled with that terrible scourge.

We have already sufficient proofs of its indigenous character, and if we would profit by this knowledge, we must give more attention to sanitary measures in order to protect ourselves from the plague.

Of the fact that it may originate in our country, we have evidence in the epidemic at Savannah in 1876, and in Memphis and New Orleans in 1879, as well as a few isolated cases in a few small towns in Arkansas, Mississippi and Louisiana. I believe Memphis and New Orleans are the only places that suffered from the disease last year that were plagued with it in 1878, this latter city only having a dozen or so of cases. This exemption can be very readily accounted for on the hypothesis of cleanliness. Those places which were so terribly scourged in 1878, with the exception of Memphis, resorted to strenuous sanitary measures, to guard against the recurrence of the disease. The Mayor of New Orleans, as early as the first of May, had raised by contributions over $100,000 to be expended in renovating and cleaning the streets and alleys, and flushing out the old canal, etc. The stagnant water in the canal with its accumulating filth for years, were removed, the canal flushed out and refilled with fresh water. All this work, of course, cost money, but its fruits resulted in ten-fold benefit to the inhabitants; and so the same may be said of other cities and towns where yellow fever prevailed in 1878.

As to Memphis, it was without city government, and, of course, no systematic plan of sanitary measures were adopted to guard it against the recurrence of the plague.

I believe it has not been said by the most strenuous advocate of the exotic origin of yellow fever, that it was imported into either New Orleans or Memphis during the year 1879. Some go so far as to say that the germs of the disease lived through the winter in a dormant or hibernated condition, and were vivified by returning warm weather. If either the hypothetical view of importation or hibernation could be true, as it respects Memphis, it must be the latter, as the disease occurred there several weeks before the few cases occurred in New Orleans. The first case in Memphis occurred on the 9th of July in the person of Mulbrandon. It is said that the disease existed in his house in 1878; and thence the idea of the dormant germ.

There are so many theories in regard to the origin and spread of yellow fever, it is a hard matter for one to keep posted, much more to believe all of them. Where there are so many different views and opinions regarding any one thing, it is pretty strong proof that it is either poorly understood, or that the subject is somewhat obscure and troublesome to investigate. But in the present instance I believe, notwithstanding the many phases of opinion, all agree that filth, if not a generator, is a great promoter of the disease. Then, this being an admitted factor in developing yellow fever by all the theorists, why should not cleanliness be made a *sine qua non* in all sections of the country where the disease is likely to prevail?

Although admitting the local origin of yellow fever, I cannot believe in its being contagious from person to person, or that it is susceptible of being communicated by means of clothing, bedding, etc.

If the theory of its production by filth be true, which, as before remarked, I have no doubt, it would not necessarily follow that the disease itself would be contagious. The poisonous element emanating from the filth enters the body, in all probability, through the respiratory organs, and produces its deleterious effects on the circulatory and nervous systems, and in due time we have the disease developed. But admitting its contagious character, would it be reasonable to suppose that a case being communicated from personal contagion would be similar in all its symptoms with one originating from filth, the other from personal effluvia? In other words, we would have the same disease with two separate and distinct causes. This, in my estimation, would be an unnatural supposition. In order to believe this proposition, we must conclude that the filth-germ can reproduce itself in the system of the patient, and thus perpetuate itself ad infinitum.

But this doctrine does not comport with the old-time maxim that similar causes produce similar results. It would be a difficult matter to believe that the effluvia from a pile of filth in the street, and the effluvia from the body of a patient, could produce similar deleterious effects on the human system, and they would alike be contagious. The seeds being essentially different in their origin and character, should, according to natural laws, produce different and unlike results; or, as you might say, distinct and unlike crops. The product of the dirt pile must be distinct and very different from the product of the fever as emanating from the human body.

Then, as it respects the contagion of yellow fever, if that property of the disease exists, it must be entirely different in its effeets and characteristics from any other known contagious disease. It is admitted by all that it is not communicable except under certain and peculiar circumstances.

In the first place, it cannot exist except in warm weather, and then only in localities where the sanitary condition is very bad. There must be a congenial soil in which to germinate its seeds, or there will be no crop. There is no other contagious disease possessing these peculiarities. Then we have no evidence that one attack will guard against a recurrence of the disease.

To be sure the climatic influences amount to something, in the way of prophylaxis, but there is no proof that a person having had the disease in the South, and changing his residence to the North for several years, would be any more exempt from attack, should he return South duiing an epidemic, than any one who had never experienced its effects.

This entirely varies fiom all contagious diseases, except those depending on contact for their propagation. I am speaking of the general law of contagious diseases, wherein one attack protects against a recurrence of the disease. Of course, there are exceptions now and then to all general laws.

We also frequently find yellow fever occurring in sound localities at the same time in the same town, the outbreaks being within a few days of each other, This is also contrary to the spread of known contagious diseases, as they have a certain number of days for incubation, or latency, before the development of the attack; and when they do spread, it is from a center or focus of the disease in action. I am unable to note a single characteristic of this disease which simluates any known contagious malady.

The theory that it can only be communicated through clothing, and not from the patient himself, I consider one of the most absurd ideas ever advanced by able and scientific men. It would strike the common-sense mind, to say the least, as being very unreasonable to suppose the contagious element, whatever that may be, would gather strongest after it left the body and enter the clothing or bedding of the patient. Of course, if there is any contagious principle, it must emanate from the body of the patient, and one would suppose it was as strong on its exit therefrom as it would be a week hence, transmitted a thousand miles, through clothing, etc. And yet this theory has its advocates among the most eminent of the profession, one of whom was the able and lamentable Woodworth, of the U. S. Marine Hospital Service. If this theory was true it would also conflict with the character of the contagia of other diseases, as the fresher they are from the body of the patient, the stronger they are.

The virus, even, of the small pox, exposed to the atmosphere, will, in a very short time, become inert. As before remarked, no difference what theory, either as to the origin or propagation of yellow fever, all agree in the two grand particulars, to wit : that cleanliness is an essential factor to prevent its occurrence, and that frost is its deadly enemy.

Now, to what conclusion would these two characteristics of the disease lead us? In what other diseases do we recognize these prominent characteristics as analogues? The answer is very palpable to all men who have experience in the treatment of malarial diseases. If our theoretical friends who attach so much importance to the infinitesimal germ were to give us all the necessary precautions, in a sanitary point of view, to ward off malarial troubles, they could not do so more accurately and precise than they do in order that we shall escape the scourge of yellow fever. Notwithstanding all this laudable advice, they seem never once to think that all the characteristics, both as to its origin and its propagation, may be satisfactorily, as well as scientifically, accounted for in precisely the same way we account for the various acknowledged malarial diseases which prevail in our country.

But some will say, that is too severe and fatal a disease to depend simply on malaria as its cause. This objection may be answered by reciting the sudden deaths resulting from congestive or pernicious chills. This disease destroys life in a shorter time, even, than yellow fever. All will admit it is a disease dependent on malaria. Then too we might cite congestive remittent, a disease simulating very closely in its symptoms that of yellow fever. In fact, I have no doubt the one has, in many instances, been mistaken for the other. Then the objection that it is a disease too grave in its character to depend on malaria will not obtain. We may have mild cases of yellow fever as well as mild cases of remittent fever. There is no doubt, however, that it is essential that a more intense degree or character of malarious poison shall be present, to develop an epidemic of yellow fever, than is necessary to produce either intermittent or remittent fever.

I am impelled to the opinion that it is good philosophy to account for all phenomena on the most common sense principles, where it can be done; and where it cannot, then we may be allowed to hypothecate some theory, no matter if it is a little metaphysical. But I am fully aware how hard it is to give up preconceived notions, but when we can do so for more rational views, I think it is our duty, and receive due credit for honest convictions.

So far as the theory of exotic origin of yellow fever is concerned, Dr. Woodhull was fully satisfied of its error, as far as it pertained to Savannah in 1876, notwithstanding his preconceived opinion to the contrary.

Our able brother, Dr. Green, late President of the Georgia Medical Society, believes the yellow fever germ acts in conjunction with the malarial germ in developing the disease, which is really equivalent to the general opinion that it is essential that certain bad sanitary conditions should exist, in order that the fever germ may take root and develop the disease. This is an acknowledgement on the part of the Doctor in the right direction.

